# Exploring the Role of Oral Microbiota in the Pathophysiology and Treatment of Bruxism

**DOI:** 10.1096/fj.202502015R

**Published:** 2025-09-02

**Authors:** Kyle Morris, Karima Ait‐Aissa, Amal M. Sahyoun, Qi Wang, Ammaar H. Abidi, Modar Kassan

**Affiliations:** ^1^ College of Dental Medicine Lincoln Memorial University, LMU Tower Knoxville Tennessee USA

## Abstract

Bruxism is an involuntary condition involving grinding and clenching of the teeth, occurring during both wakefulness and sleep. This behavior can lead to various detrimental effects on oral health, including significant tooth wear and damage, temporomandibular disorders (TMD), tooth sensitivity, gum recession, and persistent headaches along with ear pain or tinnitus. The underlying causes of bruxism have long been debated, with the consensus suggesting that psychological, genetic, and environmental factors contribute to its development. Traditionally, the etiology of bruxism has been linked to stress, anxiety, malocclusion or dental misalignment, and other behavioral factors. However, recent research has begun to explore the potential role of oral microbiota in the pathophysiology of bruxism. Emerging studies propose that disruptions in the balance of oral bacteria may influence the onset and severity of bruxism, possibly by affecting inflammatory processes or neurological pathways related to muscle function. This literature review aims to explore this novel connection, summarizing key study findings, examining the implications for treatment, and evaluating the potential mechanisms by which oral microbiota may impact bruxism. Understanding this relationship could open new avenues for therapeutic strategies targeting microbial factors in managing bruxism.

## Graphical abstract

Bruxism, characterized by involuntary teeth grinding and clenching during wakefulness and sleep, leads to oral health issues such as tooth wear, temporomandibular disorders (TMD), gum recession, and chronic pain. While traditionally attributed to psychological and behavioral factors, recent research suggests a potential link between oral microbiota imbalance and bruxism pathophysiology. This graphical abstract summarizes bruxism’s clinical manifestations, multifactorial causes, and highlights emerging evidence implicating microbial influence through inflammatory and neuromuscular pathways.

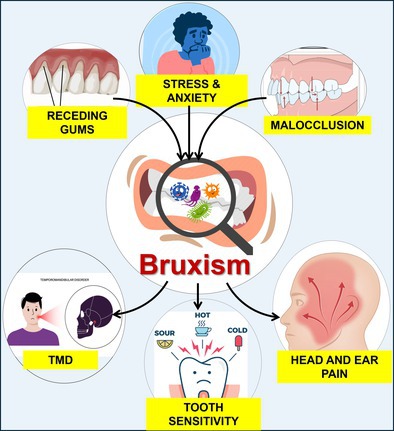

## 1 Introduction

Bruxism, defined as the repetitive grinding, gnashing, or clenching of teeth, is a common yet frequently underdiagnosed condition that affects individuals worldwide [1]. It is often associated with a variety of adverse effects, including dental wear, temporomandibular joint (TMJ) disorders, muscle pain, and headaches/migraines [1, 2]. However, beyond these well‐documented consequences, growing evidence suggests that bruxism may also have significant implications for oral health by influencing the oral microbiota, the complex community of microorganisms residing in the oral cavity [3, 4]. This interaction between bruxism and the oral microbiome is an emerging area of research, yet its potential role in both the progression of bruxism‐related symptoms and the development of oral diseases remains poorly understood.

The oral microbiota plays a crucial role in maintaining oral health by contributing to the balance of microbial communities that help prevent pathogenic overgrowth [5]. However, disturbances to this balance, such as those caused by changes in oral environmental factors or mechanical forces, have been linked to a variety of oral diseases, including periodontal disease, dental caries, and soft tissue infections [6, 7, 8, 9]. In the case of bruxism, the continuous mechanical pressure exerted on the teeth and surrounding structures may lead to physical alterations in the oral environment, such as microtrauma to gingival tissues, enamel wear, and changes in salivation, that could, in turn, impact the composition and diversity of the oral microbiota [10]. Furthermore, the stress often associated with bruxism has been implicated in influencing microbial imbalances, leading to an overgrowth of pathogenic species or a decrease in beneficial microorganisms [11].

Recent studies have begun to explore how bruxism may modulate the oral microbiome, but much remains to be uncovered regarding the precise mechanisms by which this interaction occurs [3]. For instance, bruxism‐induced alterations in the mechanical properties of the oral cavity, that lead to changes in occlusal contact, and increased wear and damage on and around the teeth, could create microenvironments that favor the growth of specific bacterial or fungal species. Research indicates a strong positive correlation between high cortisol (stress hormone) and bruxism, factors in certain microbial species involved in periodontitis including *Fusobacterium* species and *P. gingivalis* [12]. Additionally, bruxism‐related conditions such as temporomandibular joint dysfunction (TMD) or sleep disturbances could indirectly influence microbial populations through their impact on salivary flow, pH levels, and immune responses [13, 14, 15, 16, 17, 18]. Conversely, the state of the oral microbiota could also influence the development and progression of bruxism, potentially through microbial‐induced inflammation or alterations in local tissue health.

The objective of this literature review is to critically examine the current body of research on the relationship between bruxism and oral microbiota. By synthesizing existing studies, this review aims to explore the potential mechanisms through which bruxism may influence the oral microbiome and the clinical implications of these interactions. In doing so, we seek to provide a more comprehensive understanding of how changes in the oral microbiota may exacerbate the symptoms of bruxism or contribute to the development of oral diseases. Additionally, this review will highlight the need for further research to explore therapeutic interventions that could address both the mechanical and microbial aspects of bruxism, potentially leading to more effective and holistic treatment strategies.

## 2 Multifactorial Etiology of Bruxism: Psychological, Physical, and Lifestyle Influences

**FIGURE 1:**
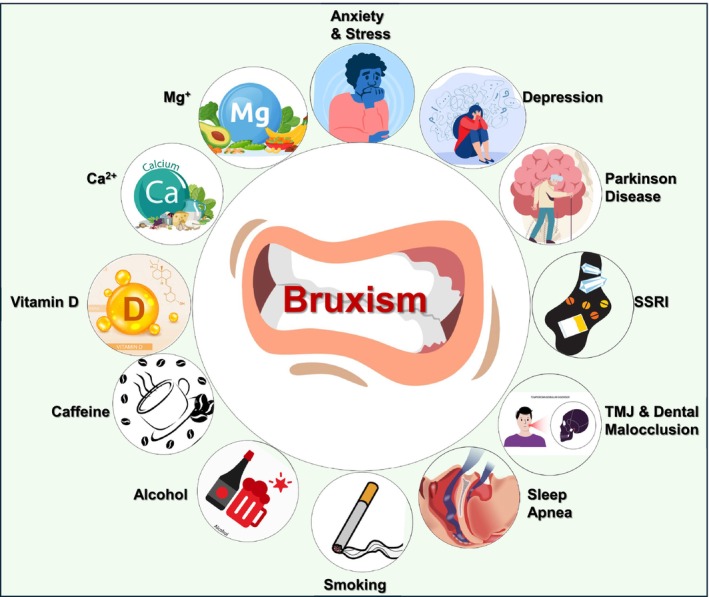
Bruxism is influenced by interconnected factors. Psychological factors include stress, anxiety, and depression, leading to unconscious grinding or clenching. Sleep disorders, such as sleep apnea and insomnia, are linked to teeth clenching during poor‐quality sleep. Physical factors involve dental malocclusion and TMJ disorders, causing jaw discomfort and uneven bites. Neurological conditions, like Parkinson's and Huntington's diseases, and pharmacological factors, such as SSRIs and antipsychotics, disrupt neurotransmitter balance and motor control. Lifestyle factors, including caffeine, alcohol, smoking, and recreational drug use, exacerbate stress and sleep disruptions. Nutritional deficiencies, such as magnesium, calcium, and vitamin D, contribute to muscle cramps and jaw tension.

Bruxism is a multifactorial condition influenced by a variety of factors that often interact in complex ways [1]. Psychological factors, such as stress, anxiety, and depression, are some of the most well‐established triggers for bruxism, with individuals experiencing high levels of emotional tension often grinding or clenching their teeth unconsciously, particularly during sleep or periods of distress [19, 20]. Sleep disorders, including sleep apnea and insomnia, are also closely linked to bruxism, as individuals may clench their teeth in response to interrupted or poor‐quality sleep [21, 22]. Physical factors, such as dental malocclusion (misalignment of teeth) and TMJ disorders, contribute to bruxism by creating an uneven bite or discomfort in the jaw, prompting unconscious grinding as the body attempts to alleviate pressure or instability [23]. Neurological conditions, including Parkinson's and Huntington's disease, along with medications that affect neurotransmitters, such as selective serotonin reuptake inhibitors (SSRIs) or antipsychotic drugs, can increase the likelihood of bruxism due to their influence on neurotransmitter levels, like dopamine disrupting the balance between serotonin and dopamine, motor control, and muscle function [24, 25, 26]. Lifestyle factors such as excessive caffeine or alcohol consumption, smoking, and recreational drug use can exacerbate stress and disrupt sleep patterns, further promoting bruxism [27, 28]. Additionally, bruxism can be influenced by age, with children often experiencing the condition, though it may persist or develop in adults under stress or due to lifestyle factors [29, 30]. Nutritional deficiencies, such as magnesium, calcium, or vitamin D, can contribute to muscle cramps and jaw tension, further exacerbating the condition [31, 32]. In conclusion, bruxism is multifactorial and can be influenced by a combination of psychological, physical, neurological, pharmacological, and lifestyle‐related factors. Identifying and addressing these underlying causes through strategies like stress management, dental correction, or medication adjustments is crucial for reducing the severity and frequency of bruxism episodes, offering the potential for more effective interventions and management of the condition (Figure 1).

## 3 Stress, Anxiety, Depression, and Their Connection to Bruxism and Oral Microbiota

Stress, anxiety, and depression have long been recognized as significant psychological factors contributing to the onset and exacerbation of bruxism [19]. Individuals experiencing high levels of emotional distress often unconsciously engage in teeth grinding or clenching, particularly during periods of sleep or heightened tension [1]. Chronic stress, in particular, has been shown to affect both the nervous and musculoskeletal systems, leading to muscle tension and increased bruxism activity [33]. Anxiety and depression further complicate(s) this dynamic by intensifying physical manifestations of emotional states, such as heightened muscle tension, disrupted sleep, and a tendency to grind teeth in response to these stresses [20]. Importantly, these psychological factors can also influence the body's immune and inflammatory responses, potentially contributing to microbial imbalances in the oral cavity [34]. The oral microbiota, a complex community of microorganisms, plays a critical role in maintaining oral health, and its delicate balance can be easily disrupted [35]. Bruxism‐induced mechanical forces, including microtrauma to the gingiva and enamel wear, can create an environment conducive to the overgrowth of pathogenic bacteria, further exacerbating oral health issues [36]. Additionally, alterations in salivation, pH levels, and immune function, often associated with chronic stress, may create favorable conditions for pathogenic species, potentially leading to diseases such as periodontal disease and dental caries [6]. Emerging evidence suggests that the physiological effects of stress, anxiety, and depression influence the oral microbiome not only by increasing the risk of bruxism but also by modulating microbial communities in ways that can complicate oral health [10]. Bruxism‐related conditions, such as temporomandibular disorder (TMD) dysfunction [37, 38, 39], may indirectly affect microbial populations by altering salivary composition and flow [15]. Thus, the relationship between bruxism and oral microbiota forms a complex, bidirectional interaction where psychological factors contribute to both the mechanical forces of bruxism and the microbial imbalances in the oral cavity, potentially exacerbating the condition and its consequences on oral health.

The composition of the oral microbiota demonstrated significant differences between individuals with depression and the healthy control group. Notably, the depression group exhibited a substantial increase in the relative abundance of *Pseudomonas* and *Capnocytophaga*, whereas *Streptococcus, Leptotrichia*, and *Solobacterium* were markedly reduced in comparison to the control group [40]. Additionally, genera such as *Faecalibacterium, Ruminococcus*, and *Solobacterium*, as well as the Lachnospiraceae family, were more prevalent in saliva samples from healthy controls [40]. In contrast, Actinobacillus and Tyzzerella, along with the Pasteurellaceae family, were more abundant in individuals with depressive symptoms [40] and may indicate a shift toward oral dysbiosis, potentially contributing to heightened inflammation, periodontal disease risk, and broader systemic health implications.

Numerous preclinical and clinical studies have demonstrated that psychological stress can impact the oral microbiome's composition [10]. It has been shown that bacteria such as *Prevotella*, *Neisseria, Haemophilus*, and *Fusobacterium* appear to play a role in these changes. For instance, the levels of *Prevotella* and *Neisseria* were found to be lower in individuals with conditions like distress and depression [41, 42]. A decrease in *Haemophilus* was noted in people with depression [41], but interestingly, elevated levels were found in those under stress [43]. A comparable pattern was observed with *Fusobacterium*, which was less abundant in depressed individuals [43], whereas *F. nucleatum* was more prevalent in people who had recently undergone a stressful experience [44]. Additional studies have reported an increase in the abundance of the phylum Spirochaetes and the order *Spirochaetales* in individuals with higher depression levels, with these taxa showing a positive correlation to anxiety and depression symptoms [45]. In a human study, salivary levels of *Streptococcus gordonii* dropped significantly just before an academic examination compared to samples collected weeks later, suggesting a link between acute stress and microbial shifts [46]. Finally, in rats subjected to chronic stress, *Facklamia* increased while *Corynebacterium* decreased compared to control rats [47].

Several studies have reported that *Prevotella* is increased in patients with anxiety and depression [48, 49]. A negative correlation between *Gemella* abundance and depression has also been identified [50]. Research on Lachnospiraceae suggests that it may contribute to the development of depression by influencing the host's inflammation levels [51]. Additionally, a genetic study found a connection between the oral microbiome, including *Granulicatella*, and depression [52]. *Veillonella* has also been implicated, with altered levels observed in both the gut and oral microbiotas of individuals diagnosed with depression [49]. In addition, increased levels of *Selenomonas* have been observed in patients with depression and anxiety [53].

A study by Deng et al. [3] found differences in the oral microbiota composition between a control group and a bruxism group. The data revealed that *Streptococcus, Gemella*, and *Granulicatella* were less abundant in individuals with bruxism, while *Catonella, Oribacterium, Johnsonella, Lachnospiraceae, Prevotella, Veillonella*, and *Slenomonas* showed increased abundance in the bruxism group [3].

**FIGURE 2:**
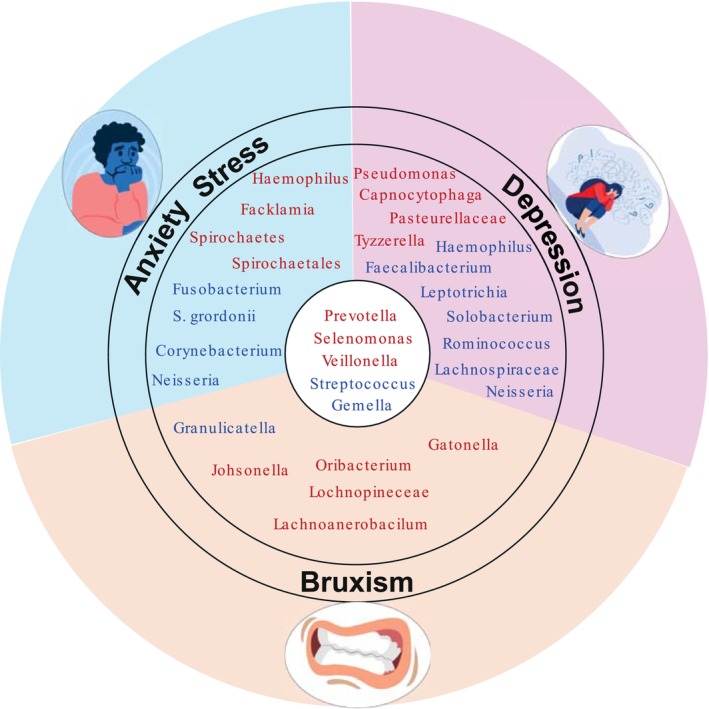
Oral microbiota variations and similarities associated with depression, bruxism, anxiety, and stress. Common bacterial changes across all conditions include increased Prevotella, Selenomonas and Veillonella, and decreased Streptococcus and Gemella, suggesting shared dysbiotic patterns highlighting the complex interplay between oral microbiota, bruxism, mental health, and stress‐related conditions. Bacteria highlighted in Red indicate those that are increased in abundance, while bacteria highlighted in Blue indicate those that are reduced in abundance.

These findings highlight the overlap of certain bacteria between psychological conditions like depression and anxiety, and bruxism, suggesting that psychological factors may influence bruxism via the oral microbiome (Figure 2).

## 4 Sleep Disorders, Bruxism, and Their Impact on Oral Microbiota

Sleep disorders, such as sleep apnea and insomnia, have been closely linked to bruxism and may also influence the composition and health of the oral microbiota [54, 55]. Sleep apnea, a sleep‐related breathing disorder characterized by recurrent episodes of partial or complete airway obstruction (obstructive sleep apnea) or impaired respiratory drive (central sleep apnea) during sleep, results in intermittent hypoxia and sleep fragmentation [56]. These physiological disruptions can trigger compensatory neuromuscular responses, including the unconscious grinding or clenching of teeth (sleep bruxism), as the body attempts to stabilize airway patency and oxygen levels, which can contribute to significant dental wear and damage, as well as to the alteration of the oral environment [6]. Insomnia, another common sleep disorder marked by difficulty falling or staying asleep, has been linked to bruxism as individuals may experience heightened stress, anxiety, and muscle tension, all of which contribute to teeth grinding [57, 58, 59]. The lack of restful sleep further exacerbates these effects by increasing fatigue and exacerbating daytime stress, creating a vicious cycle of bruxism and sleep disruption.

Beyond the mechanical impacts of bruxism, sleep disorders may also affect the oral microbiota. Sleep apnea, for example, can lead to dry mouth (xerostomia) due to mouth breathing during episodes of airway obstruction, which reduces the mouth's ability to self‐cleanse and maintain an optimal microbial balance [60, 61]. This can lead to an overgrowth of pathogenic microorganisms, contributing to oral health problems such as gingivitis, periodontal disease, and dental caries [62, 63]. Insomnia and fragmented sleep have also been shown to impact salivation and immune function, further compromising the oral cavity's defense mechanisms against harmful bacteria [64]. Additionally, chronic stress and anxiety, often associated with sleep disorders, can influence microbial imbalances by promoting inflammation and altering the pH of the oral environment, creating conditions that favor the growth of oral pathogens [6].

Emerging research is beginning to examine the relationship between sleep disorders, bruxism, and the oral microbiome, though much remains to be understood. The connection between these factors is likely bidirectional, with sleep disturbances not only promoting bruxism but also influencing the microbial composition of the oral cavity, which in turn may exacerbate the effects of bruxism on oral health. As such, a more comprehensive understanding of how sleep disorders impact bruxism and oral microbiota could lead to novel treatment approaches that address both the mechanical and microbial aspects of these interrelated conditions.

Studies have shown that the oral microbiota of patients with obstructive sleep apnea (OSA) differs significantly from that of individuals without the condition [65]. Additionally, the nasal microbiome in individuals with severe OSA has been found to be altered, with an increased abundance of bacteria such as *Prevotella* and *Veillonella* [66]. Another study found that *Gemella* species were associated with a healthy oral microbiome, whereas *Cutibacterium*, *Propionibacterium*, and *Bifidobacterium* species exhibited a strong correlation with OSA [67]. Similarly, an increased abundance of Ruminococcaceae and Lachnospiraceae was directly correlated with OSA patients [68]. Conversely, a separate study reported a reduction in *Peptostreptococcus*, *Alloprevotella*, and *Granulicatella* in the saliva of individuals with OSA [69] suggesting potential microbial imbalances associated with the condition.

A study conducted by Deng et al. [3] revealed significant differences in the oral microbiota composition between individuals with bruxism and a control group. The study showed that bacteria such as *Streptococcus, Gemella*, and *Granulicatella* were found in lower abundance in the bruxism group. In contrast, *Catonella, Oribacterium, Johnsonella, Lachnospiraceae, Prevotella, Veillonella*, and *Slenomonas* were more abundant in individuals with bruxism [3].

**FIGURE 3:**
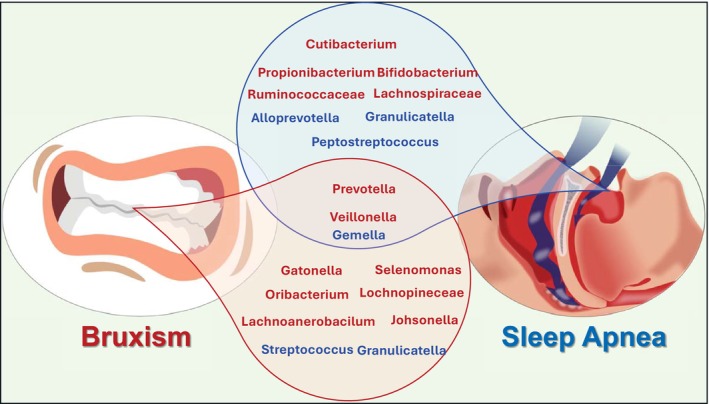
Oral microbiota variations and similarities associated with Sleep Apnea and bruxism. Common bacterial changes across all conditions include increased Prevotella and Veillonella, and decreased Gemella, suggesting shared dysbiotic patterns highlighting the complex interplay between oral microbiota, bruxism and sleep apnea. Bacteria highlighted in red indicate those that are increased in abundance, while bacteria highlighted in blue indicate those that are reduced in abundance.

The oral microbiota in individuals with obstructive sleep apnea and bruxism exhibits a notable shift toward anaerobic and Gram‐negative bacterial species. This microbial transition, characterized by increased abundance of genera such as *Prevotella* and *Veillonella*, reflects a dysbiotic environment that may contribute to local inflammation and altered immune responses within the oral cavity (Figure 3).

## 5 The Influence of Dental Malocclusion and TMJ Disorders on Bruxism and Oral Microbiota

Dental malocclusion and TMJ disorders are significant factors contributing to bruxism and can have a profound impact on the oral microbiota. Bruxism‐related conditions, such as TMJ dysfunction, may indirectly affect microbial populations by altering salivary composition and flow [15]. Malocclusion refers to improper alignment of the maxillary and mandibular teeth, creating uneven occlusal contacts and force distribution during functional activities [70, 71]. Over time, this may contribute to parafunctional habits, including involuntary bruxism, particularly during sleep. Bruxism, in turn, exacerbates the mechanical stress on the teeth and surrounding structures, potentially contributing to enamel wear, tooth fractures, and discomfort in the TMJ [1].

TMJ disorders encompass a range of conditions characterized by dysfunction, inflammation, or structural abnormalities of the TMJs, which articulate the mandible with the cranial base [72]. They are frequently associated with bruxism, as affected individuals may clench or grind their teeth in response to joint pain or restricted movement, further aggravating TMJ dysfunction [73]. This response can further aggravate both the TMJ and the surrounding muscles, creating a cycle of discomfort and dysfunction.

Both dental malocclusion and TMJ disorders not only contribute to the physical manifestations of bruxism but also have an impact on the oral microbiota [74, 75, 76]. Bruxism‐related mechanical forces, such as the grinding of teeth, can lead to microtrauma of the gingival tissues and the enamel surface, creating an environment that is conducive to the proliferation of pathogenic bacteria [6, 7, 77]. The wear on tooth enamel can expose the underlying dentin, which may be more susceptible to microbial colonization due to structural composition, permeability, and lack of protective mineralization [77, 78]. Additionally, alterations in salivation patterns and the increased likelihood of gum recession associated with bruxism and TMJ disorders may contribute to changes in the oral environment, affecting the diversity and balance of microbial communities [3, 7].

Sensitivity in teeth or gingiva due to malocclusion or TMJ dysfunction may also affect oral hygiene behavior, such as food avoidance or inconsistent brushing, further promoting [79, 80]. Furthermore, both malocclusion and TMJ disorders can disrupt normal bite forces, which reduce maximal opening of the mouth or vertical dimension, which in turn may affect the mechanical cleaning of the teeth and gingiva during chewing/brushing [81, 82, 83], potentially reducing the mouth's natural ability to maintain a healthy microbiota.

Recent studies suggest that there is a bidirectional relationship between oral health and bruxism, with malocclusion and TMJ dysfunction [84, 85] potentially influencing the oral microbiome and vice versa. The disruption in microbial balance caused by bruxism may, in some cases, exacerbate the severity of dental malocclusion and TMJ symptoms by promoting inflammation, increasing tooth sensitivity, and affecting the healing processes of damaged tissues. As such, addressing both the mechanical and microbial factors in individuals with bruxism may provide a more holistic approach to managing these conditions and improving overall oral health.

In conclusion, dental malocclusion and TMJ disorders play a crucial role in the development and progression of bruxism, which in turn can significantly impact the oral microbiota. The interaction between these factors highlights the need for comprehensive treatment strategies that address both the mechanical aspects of misalignment and joint dysfunction, as well as the microbial imbalances that may result from bruxism. Further research into the mechanisms by which these conditions affect oral health could help develop more effective therapeutic approaches for individuals suffering from bruxism, malocclusion, and TMJ disorders.

A recent study showed several bacteria such as *Coprobacter*, *Ruminococcus torques* group, *Catenibacterium*, *Lachnospiraceae*, *Turicibacter*, *Victivallis*, *Methanobacteriales*, *Methanobacteriaceae*, and *Methanobacteria* were identified as a trigger for TMJ [86]. Another study showed a positive correlation between TMJ and *Veillonella* [87]. A study by Deng et al. examined the differences in oral microbiota composition between individuals with bruxism and a control group [3]. The findings revealed a decreased abundance of *Streptococcus*, *Gemella*, and *Granulicatella* in the bruxism group, whereas *Catonella*, *Oribacterium*, *Johnsonella*, Lachnospiraceae, *Prevotella*, *Veillonella*, and *Selenomonas* were more prevalent among individuals with bruxism [3]. These findings emphasize the connection between TMJ conditions and bruxism, suggesting that the impact of TMJ on bruxism may be mediated through changes in the oral microbiome.

Malocclusion, caused by misalignment of the upper and lower teeth, creates an unhealthy environment for the oral microbiome. This imbalance raises the risk of dental caries and periodontal diseases. Malocclusion can also lead to dysbiosis in the salivary microbiome, further affecting oral health [88, 89]. It cannot be ruled out that the observed microbial differences may be due to variations in oral hygiene practices associated with fixed orthodontic appliances, rather than malocclusion itself.

A study by Yamashita et al. [90] identified a correlation between shifts in salivary microbiota composition and oral health conditions such as dental caries and periodontal disease. The study found an increased abundance of *Prevotella histicola* , *Prevotella melaninogenica* , *Veillonella parvula* , *Veillonella atypica* , *Streptococcus salivarius* , and *Streptococcus parasanguinis* , while *Neisseria flavescens* , *Haemophilus parainfluenzae* , *Porphyromonas pasteri*, *Gemella sanguinis* , and *Granulicatella adiacens* were reduced in individuals with these conditions.

**FIGURE 4:**
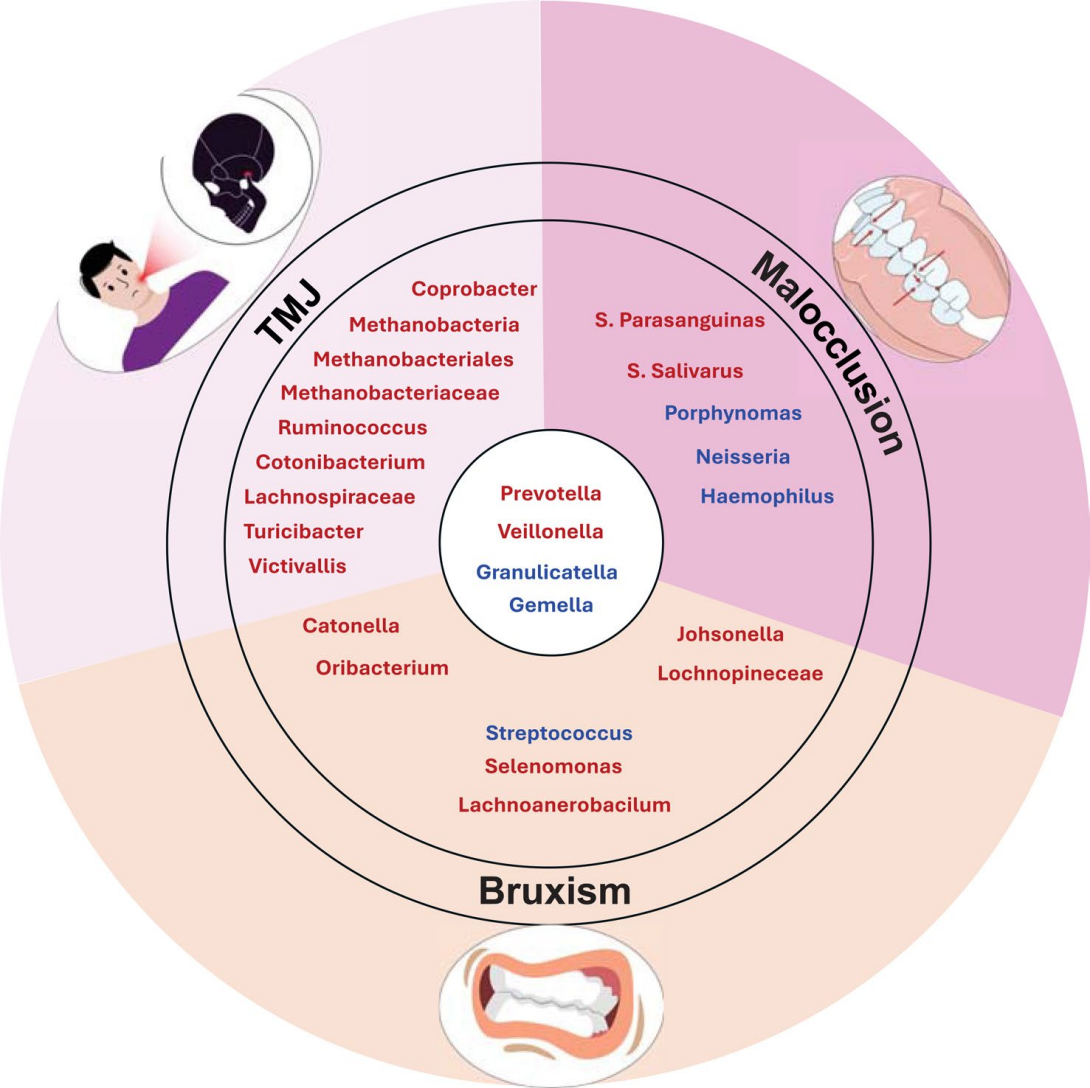
Oral microbiota variations and similarities associated with TMJ, malocclusion and bruxism. Common bacterial changes across all conditions include increased Prevotella and Veillonella, and decreased Granulicatella and Gemella, suggesting shared dysbiotic patterns highlighting the complex interplay between oral microbiota and TMJ, malocclusion and bruxism. Bacteria highlighted in red indicate those that are increased in abundance, while bacteria highlighted in blue indicate those that are reduced in abundance.

The oral microbiome in conditions such as TMJ dysfunction, bruxism, and malocclusion demonstrates a distinct shift toward a Gram‐negative, anaerobic‐dominated profile, reflecting a state of dysbiosis. Health‐associated, Gram‐positive facultative anaerobes like *Streptococcus* and *Gemella* are notably reduced, while pro‐inflammatory anaerobes such as *Prevotella*, *Veillonella*, and members of the Lachnospiraceae family are elevated. Additionally, the detection of archaea like *Methanobacteria* suggests increasingly hypoxic conditions within the oral cavity, likely exacerbated by chronic parafunctional habits or altered salivary dynamics. These microbial shifts not only mirror the inflammatory and mechanical stressors of these conditions but may also play an active role in perpetuating tissue damage and impaired healing (Figure 4).

## 6 Impact of Lifestyle Factors on Bruxism and Oral Microbiota: A Bidirectional Relationship

Lifestyle factors, including excessive caffeine and alcohol consumption, smoking, and recreational drug use (such as methamphetamine) [91], can significantly influence the development and progression of bruxism, while also affecting the balance of oral microbiota. Caffeine, commonly associated with stress and anxiety, may increase muscle tension and disrupt sleep, contributing to both daytime and nocturnal bruxism [92, 93]. Similarly, while alcohol initially induces relaxation by enhancing GABAergic activity, it later disrupts sleep quality and may increase nighttime muscle activity, promoting bruxism [94, 95, 96, 97]. Smoking, on the other hand, has been associated with a heightened risk of periodontal disease and oral infections, potentially complicating the oral environment in individuals with bruxism [98, 99, 100]. Additionally, recreational drug use can increase the likelihood of bruxism due to its impact on both the nervous system and muscle control [28]. These lifestyle behaviors not only intensify mechanical forces on the dentition but also alter salivary flow, pH, and immune defenses, promoting microbial imbalance. Bruxism‐related microtrauma may further disrupt the oral barrier, facilitating colonization by pathogenic bacteria and worsening oral health.

A recent study in the Korean population revealed that *Oribacterium*, *Campylobacter*, and *Megasphaera* were abundant in coffee consumers, whereas *Saccharimonadales*, *Clostridia*, and *Catonella* were abundant in alcohol drinkers. They found increased levels of *Stomatobaculum* in the saliva of smokers, compared with that of non‐smokers [101]. Another study showed that smokers, when compared to non‐smokers, had increased levels of *Veillonella*, *Prevotella*, and *Leptotrichia*, and lower levels of *Gemella, Neisseria, Streptococcus*, and *Haemophilus* [102].

Furthermore, alcohol consumption showed decreased levels of oral *Streptococcus, Lactococcus*, and *Lactobacillus* and increased levels of *Prevotella*, *Helicobacter, Proteobacteria, Alloprevotella*, and *Janthinobacterium* [103]. Higher levels of *Prevotella, Porphyromonas*, and *Bacteroides* were found in heavy coffee drinkers in a study of a group of 147 individuals [104].

Previous studies have shown that drug abuse, such as consuming methamphetamine, induced alteration in oral microbiota [105, 106]. Yang Yu et al. showed that methamphetamine users exhibited higher relative abundance in several bacterial taxa that were known to be related to oral diseases in saliva microbiota [105]. To be more specific, they showed an increased level of *Veillonella, Selenomonadales*, and *Prevotella* and a decrease in Neisseria in methamphetamine users compared to healthy individuals [105]. Another study showed that *Streptococcus* and *Leptotrichia* were decreased and *Porphyromonas* was increased in methamphetamine users compared to healthy individuals [106].

A study by Deng et al. [3] examined the oral microbiota composition in individuals with bruxism compared to a control group. The findings revealed that bacteria like *Streptococcus, Gemella*, and *Granulicatella* were less abundant in those with bruxism. In contrast, species such as *Catonella, Oribacterium, Johnsonella, Lachnospiraceae, Prevotella, Veillonella*, and *Selenomonas* were found to be more prevalent in the bruxism group [3].

**FIGURE 5:**
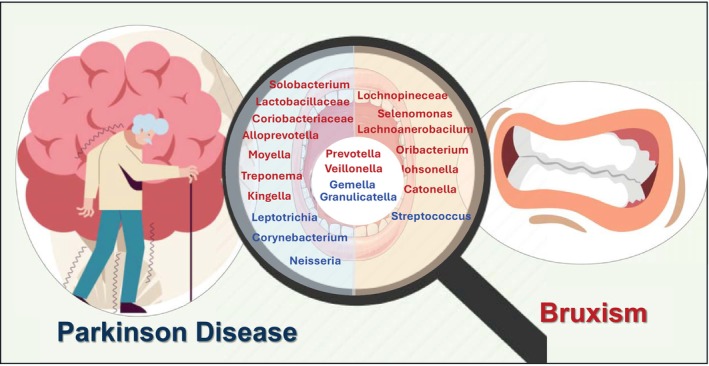
Oral microbiota variations and similarities associated with bruxism and lifestyle factors. Common bacterial changes across all conditions include increased Prevotella, selenomonas, oribacterium and Veillonella, and decreased Streptococcus and Gemella, suggesting shared dysbiotic patterns highlighting the complex interplay between oral microbiota brusims and lifestyle factors such as smoking, alcohol, and coffee drinkers and drug use. Bacteria highlighted in red indicate those that are increased in abundance, while bacteria highlighted in blue indicate those that are reduced in abundance.

Lifestyle habits such as smoking, alcohol consumption, coffee intake, and methamphetamine use significantly alter the oral microbiome, driving a shift toward Gram‐negative, anaerobic, and pro‐inflammatory bacteria like *Prevotella*, *Veillonella*, *Porphyromonas*, and *Selenomonadales*. Concurrently, beneficial Gram‐positive aerobes and facultative anaerobes such as *Streptococcus*, *Neisseria, Gemella*, and *Haemophilus* are reduced. These microbial imbalances mirror patterns seen in bruxism, suggesting a shared dysbiotic signature among behavioral stressors and lifestyle‐related exposures that may contribute to inflammation, oral disease, and tissue breakdown (Figure 5).

## 7 Neurological Conditions, Pharmacological Mechanisms, and Their Impact on Bruxism and Oral Microbiota

Neurological conditions such as Parkinson's disease and Huntington's disease, along with medications that affect neurotransmitter function, have been linked with the onset of bruxism [24, 107, 108, 109], which may subsequently influence the composition and balance of the oral microbiota. In Parkinson's disease, dopaminergic dysfunction can impair motor control of jaw muscles, contributing to bruxism [1, 110]. Similarly, Huntington's disease causes involuntary, unpredictable, and irregular muscle movements known as chorea, which can contribute to bruxism due to impaired motor coordination and control [1]. These neurological conditions often lead to chronic, uncontrolled bruxism, which may result in significant dental wear and TMJ discomfort [1].

The connection between bruxism and oral health is especially significant when evaluating its effects on the oral microbiota. The repetitive mechanical stress from teeth grinding and jaw clenching can lead to microtrauma in the enamel and surrounding gingival tissues [80]. These alterations in the oral environment may disrupt microbial balance, fostering conditions that encourage the proliferation of pathogenic bacteria. Furthermore, individuals with neurological conditions may experience difficulty with oral hygiene due to motor impairments or muscle rigidity, which can lead to a reduction in the mouth's natural defenses against harmful microorganisms [111]. This increased risk of microbial imbalance can contribute to oral diseases such as periodontal disease, dental caries, and soft tissue infections.

Medications that affect neurotransmitters, such as selective serotonin reuptake inhibitors (SSRIs) and antipsychotic drugs, have also been linked to bruxism [24, 108, 112, 113, 114]. These medications, commonly prescribed for mood disorders, anxiety, and schizophrenia, can have side effects that increase the likelihood of bruxism [24, 108, 112, 113, 114]. SSRIs, for example, are known to cause serotonin imbalances, which may disrupt the normal regulation of muscle tone and increase the risk of involuntary muscle contractions, including those responsible for grinding or clenching teeth [24]. Similarly, antipsychotic medications, which also affect neurotransmitter systems, have been associated with movement disorders such as tardive dyskinesia, which can involve bruxism as a secondary symptom [115].

The side effects of these medications can exacerbate the mechanical impacts of bruxism, leading to greater wear on teeth and enamel. Moreover, both neurological conditions and medications that influence neurotransmitter function alter salivary flow, pH levels, and immune function, creating an environment in which pathogenic bacteria may thrive. Changes in salivation, which are common in patients with Parkinson's or as a result of medication side effects, can lead to xerostomia (dry mouth), further reducing the natural antimicrobial properties of saliva and facilitating the growth of harmful microorganisms in the oral cavity [116, 117]. This disruption of the oral microbiome could, in turn, contribute to the development of oral infections, inflammation, and a decrease in overall oral health.

In summary, while often overlooked, neurological conditions such as Parkinson's and Huntington's disease, along with neurotransmitter‐altering medications, not only play a role in the development of bruxism but also have profound, yet underrecognized, effects on the oral microbiota. The mechanical strain of bruxism on the teeth and gingiva, combined with altered salivation and challenges in maintaining oral hygiene, heightens the risk of microbial imbalances in the oral cavity. Further research is needed to explore the precise mechanisms through which these conditions and medications influence the oral microbiome, as well as to develop targeted strategies for managing both bruxism and its associated oral health complications.

A study showed that saliva from Parkinson's disease (PD) patients revealed increased levels of *Veillonella*, *Treponema, Kingella*, and Alloprevotella, while *Leptotrichia* levels were notably decreased compared to control saliva [118]. Another study identified significant shifts in oral microbial composition between PD patients and control subjects, highlighting an increase in potential opportunistic oral pathogens. In PD patients, Prevotella, Prevotellaceae, Veillonella, Solobacterium, Veillonellaceae, Lactobacillaceae, and Coriobacteriaceae were found in greater abundance. Conversely, Capnocytophaga, Rothia, Kingella, Leptotrichia, Actinomyces, and Leptotrichiaceae exhibited reduced levels. Additionally, other taxa showing a decrease in the PD group included Haemophilus, Neisseria, Gemella, Corynebacterium, Granulicatella, an unclassified Flavobacteriaceae OTU, Pasteurellaceae, Neisseriaceae, Micrococcaceae, Carnobacteriaceae, and Corynebacteriaceae, while Moryella and Erysipelotrichaceae were notably increased [119].

Currently, no studies have directly established a link between Huntington's disease and alterations in the oral microbiota. Research on Huntington's disease has predominantly focused on its genetic and neurological aspects, leaving its potential impact on oral health and microbial composition largely unexplored. Further studies are needed to understand whether neurodegenerative changes in Huntington's disease influence oral microbiota dynamics. Antipsychotic drugs have been studied for their effects on oral health, but there is a lack of research specifically focusing on their impact on the oral microbiota. While these medications are known to influence factors such as salivation and gum health, their direct effects on the microbial communities in the mouth remain unexplored.

A study by Deng et al. [3] examined the oral microbiota composition in individuals with bruxism compared to a control group. The findings revealed that bacteria like *Streptococcus*, *Gemelli*, and *Granulicatella* were less abundant in those with bruxism. In contrast, species such as *Catonella*, *Oribacterium*, *Johnsonella*, *Lachnospiraceae*, *Prevotella*, *Veillonella*, and *Selenomonas* were found to be more prevalent in the bruxism group [3].

**FIGURE 6:**
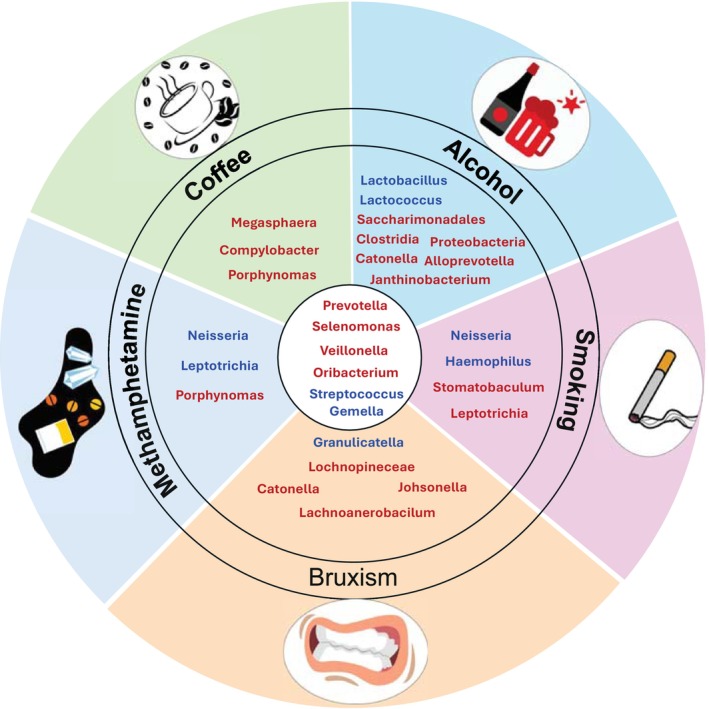
Oral microbiota variations and similarities associated with bruxism and PD. Common bacterial changes across all conditions include increased Prevotella and Veillonella, and decreased Granulicatella and Gemella, suggesting shared dysbiotic patterns highlighting the complex interplay between oral microbiota bruxism and PD. Bacteria highlighted in red indicate those that are increased in abundance, while bacteria highlighted in blue indicate those that are reduced in abundance.

Studies on patients with Parkinson's disease (PD) reveal a distinct oral microbial shift toward anaerobic and opportunistic Gram‐negative bacteria, with elevated levels of *Veillonella, Prevotella, Alloprevotella, Treponema*, and *Solobacterium*. Simultaneously, there is a marked decrease in beneficial or commensal taxa such as *Leptotrichia*, *Neisseria, Gemella, Haemophilus, Rothia*, and *Actinomyces*. These changes reflect a loss of microbial diversity and increased colonization by potential pathogens, likely influenced by PD‐related salivary dysfunction, altered immunity, and medication use. Although Huntington's disease has not yet been directly linked to oral microbiome changes, antipsychotic medications commonly prescribed for neurological disorders are known to affect oral health through changes in saliva flow and tissue integrity. Interestingly, the microbial profile of PD patients overlaps with that observed in bruxism, suggesting a possible shared dysbiotic pattern driven by neurological, behavioral, and physiological stressors (Figure 6).

## 8 Nutritional Deficiencies and Their Impact on Bruxism and Oral Microbiota

Nutritional deficiencies, particularly in magnesium, calcium, and vitamin D, have been suggested as potential contributors to bruxism and may also influence the health and balance of the oral microbiota [31, 32, 120]. Magnesium is essential for muscle function and relaxation, and its deficiency can result in muscle cramps, tension, and spasms, potentially contributing to involuntary teeth grinding or clenching [31, 121, 122]. Similarly, calcium, which is vital for muscle contraction and nerve function, plays a key role in maintaining proper muscle tone, including that of the jaw muscle [123]. Insufficient calcium levels can lead to muscle instability, increasing the likelihood of bruxism [32]. Additionally, calcium is essential for the maintenance of the integrity of tooth enamel, and a deficiency can contribute to increased tooth sensitivity and vulnerability to damage from grinding [78].

Moreover, Vitamin D, known for its role in calcium absorption and bone health, is another critical nutrient that can influence both bruxism and oral health [124]. A deficiency in vitamin D can impair the absorption of calcium, leading to weakened bones and teeth [125]. In the context of bruxism, a deficiency in vitamin D can also impair muscle function, potentially leading to increased jaw tension and teeth grinding [32]. Furthermore, Vitamin D plays a pivotal role in modulating the immune system, and its deficiency has been linked to increased inflammation in the oral cavity, potentially disrupting the balance of the oral microbiota [126]. Research indicates that vitamin D exerts anti‐inflammatory effects by influencing both innate and adaptive immune responses, which are crucial in maintaining oral health [126]. Chronic inflammation, whether from a vitamin D deficiency or other factors, can create an environment that favors the growth of pathogenic bacteria, which could lead to periodontal disease and other oral health issues [127, 128, 129].

The relationship between nutritional deficiencies and bruxism may also extend to their impact on the oral microbiome. Bruxism itself, through its mechanical forces, can create microtrauma in the gingiva and enamel, which increases the potential for microbial colonization [130]. A diet deficient in magnesium, calcium, or vitamin D may impair the body's ability to repair this damage, creating a more favorable environment for the proliferation of harmful bacteria. Additionally, nutritional deficiencies can negatively impact salivation, reducing the natural antibacterial effects of saliva and further disrupting the microbial balance in the mouth [131, 132].

Thus, addressing these nutritional deficiencies may not only help reduce the severity of bruxism but could also support the health of the oral microbiota. A case–control study found that individuals with sleep bruxism had significantly lower serum vitamin D levels and dietary calcium intake compared to controls. The study concluded that vitamin D deficiency and low calcium consumption were significantly associated with sleep bruxism [32]. Therefore, ensuring an adequate intake of magnesium, calcium, and vitamin D may help mitigate muscle tension, reduce the frequency of bruxism, and promote the healing of oral tissues affected by grinding. Additionally, proper nutrition can support the overall immune system and oral health, helping to maintain a balanced microbiome that prevents the overgrowth of harmful bacteria. As such, improving nutritional status may represent an important strategy for managing both bruxism and the associated oral health complications, while also providing a foundation for the maintenance of a healthy oral microbiota.

In conclusion, nutritional deficiencies in magnesium, calcium, and vitamin D may contribute to the onset and exacerbation of bruxism, while also impacting the oral microbiota. The interplay between nutrition, bruxism, and oral health highlights the need for a comprehensive approach to managing bruxism, one that includes addressing both the mechanical factors and the nutritional components that can influence muscle function, oral tissue health, and microbial balance. Further research is needed to fully understand the mechanisms through which these deficiencies impact bruxism and oral microbiota, as well as to explore potential dietary interventions for individuals suffering from these interrelated conditions.

## 9 Clinical Implications

The complex nature of bruxism, driven by psychological, physical, pharmacological, and lifestyle factors, requires a multifaceted treatment approach. While dental interventions such as occlusal splints are commonly used, addressing underlying factors like stress, sleep disorders, and malocclusion is essential for effective management. Incorporating strategies to reduce psychological stress, certain medications, and managing sleep disorders can help reduce the frequency and severity of bruxism episodes.

The emerging link between bruxism and oral microbiota disturbances highlights the need for dental professionals to consider the microbial aspect of bruxism‐related oral health issues. Bruxism exerts mechanical forces on oral tissues, leading to microtrauma that can create an environment conducive to the proliferation of pathogenic bacteria. This disruption in the oral microbiota may increase the risk of oral diseases such as periodontal disease and dental caries. Implementing treatments that balance the oral microbiota, including prebiotics, probiotics, and antimicrobial therapies, could potentially mitigate these risks.

Probiotics, particularly strains of *Lactobacillus*, have demonstrated antimicrobial properties against oral *Streptococcus mutans* , a primary contributor to dental caries. *Lactobacillus* produces substances like lactic acid and bacteriocins, which inhibit harmful bacteria. The clinical strategies for using prebiotics, probiotics, and antimicrobial therapies targeting specific pathogens can help restore a healthy oral microbiome, thereby reducing the incidence of oral diseases associated with bruxism‐induced microtrauma.

Nutritional deficiencies in magnesium, calcium, and vitamin D may exacerbate bruxism and its oral health consequences. Clinicians should assess the nutritional status of bruxism patients, particularly those experiencing muscle tension and jaw discomfort. Supplementation with magnesium, calcium, and vitamin D may reduce bruxism symptoms and improve overall oral health, including the maintenance of the alveolar bone, supporting enamel remineralization and a balanced oral microbiome.

A saliva‐based diagnostic test for bruxism can also be used to combine targeted qPCR analysis of disease‐associated bacteria with cytokine and cortisol profiling to assess inflammation and stress. This dual approach can help clinicians detect early signs of dysbiosis and oral‐systemic imbalance, enabling better diagnosis, monitoring, and treatment planning. Given the multifactorial nature of bruxism, collaborative care involving psychologists, sleep specialists, dentists, and nutritionists can offer comprehensive management for bruxism patients. This interdisciplinary approach can help address both the mechanical and microbial factors contributing to the condition, optimizing treatment outcomes.

## 10 Future Directions

Our review study highlights a bidirectional relationship between bruxism and the oral microbiota, where bruxism‐induced microtrauma can alter microbial composition, while specific microbial imbalances may also contribute to the development or severity of bruxism. However, more research is needed to identify the precise microbial communities involved in this interaction. Future studies should explore the mechanisms through which bruxism disrupts microbial balance and, conversely, how shifts in the oral microbiota may influence neuromuscular activity and bruxism severity. Understanding these connections could pave the way for microbial‐based interventions, such as prebiotics, probiotics, or targeted antimicrobial therapies, as potential adjuncts in bruxism treatment.

Research into the role of nutritional deficiencies in bruxism could lead to personalized treatment plans that include targeted dietary recommendations or supplements. Future studies should investigate the effectiveness of magnesium, calcium, and vitamin D supplementation in reducing bruxism symptoms and supporting oral health.

Given the role of psychological factors like stress and sleep disorders in bruxism, longitudinal studies examining the long‐term effects of managing these conditions on bruxism severity would be valuable. This could help identify the most effective approaches for reducing bruxism in individuals experiencing chronic stress or sleep disturbances.

New treatment approaches should aim to address both the mechanical effects of bruxism and the microbial imbalances in the oral cavity, as emerging evidence suggests a bidirectional relationship between oral microbiota and bruxism. Beyond conventional interventions like occlusal splints and behavioral therapies, microbial‐targeted treatments may offer additional benefits in preventing associated oral diseases such as periodontal disease, dental caries, and mucosal inflammation. Probiotic formulations, including *Lactobacillus reuteri, Bifidobacterium*, and *Streptococcus salivarius K12*, could help restore microbial balance, while oral prebiotics such as levan, arginine, xylitol, and polyphenols may promote the growth of beneficial bacteria and reduce pathogenic overgrowth. Targeted antimicrobial therapies, including chlorhexidine rinses, silver diamine fluoride, and localized antibiotic gels, could further aid in controlling microbial shifts linked to bruxism‐induced oral trauma.

Additionally, pharmacological interventions that modulate both neuromuscular activity and microbial health, such as magnesium and calcium supplementation, melatonin therapy, and cannabinoids like cannabidiol (CBD), may help regulate bruxism severity while supporting a balanced oral microbiome. By integrating these approaches, bruxism management can evolve beyond mechanical protection to a more comprehensive, microbiome‐informed strategy that mitigates both the neuromuscular and microbial consequences of the condition. Given the potential impact of bruxism on both oral health and overall well‐being, future research should focus on identifying high‐risk populations, such as those with neurological conditions or malocclusion. Preventive measures, including early interventions and education, could help reduce the burden of bruxism and its related oral health complications.

By deepening our understanding of the complex relationship between bruxism, oral microbiota, and other contributing factors, future research can lead to more holistic and effective approaches for managing bruxism and enhancing oral health.

## 11 Conclusion

Bruxism is a multifactorial condition influenced by psychological, neurological, physical, pharmacological, and environmental factors, which can have significant consequences for both oral health and overall well‐being. The emerging research highlighting the relationship between bruxism and oral microbiota adds a new dimension to our understanding of the condition. The mechanical forces generated by bruxism, including grinding and clenching, can disrupt the delicate equilibrium of the oral microbiome, creating an environment that promotes the overgrowth of pathogenic bacteria and increases the risk of oral diseases such as periodontal disease and dental caries. Additionally, factors such as stress, sleep disorders, malocclusion, and neurological conditions all influence both bruxism and the health of the oral microbiota, creating a complex, bidirectional relationship.

The evolving role of the oral microbiome in bruxism emphasizes the need for a comprehensive approach to treatment that goes beyond traditional mechanical or behavioral therapies. Addressing both the physical and microbial aspects of bruxism through integrated strategies, including nutritional interventions, stress management, and targeted microbial therapies, could lead to more effective and holistic management of the condition. Furthermore, the impact of bruxism on oral health highlights the importance of early diagnosis and intervention to prevent long‐term dental deterioration and enhance overall quality of life.

Although still in its early stages, the exploration of therapeutic approaches that address both microbial imbalances and the mechanical aspect of bruxism presents promising new avenues for research and clinical application. Future studies should focus on better understanding the mechanisms through which the oral microbiota contributes to bruxism, as well as exploring the development of targeted treatments that address both the microbial and mechanical factors involved. Ultimately, such advancements could lead to more effective, personalized treatment options for individuals affected by bruxism, enhancing both their oral and overall health.

## 12 Limitations

While this review provides a comprehensive analysis of bruxism, certain limitations must be acknowledged. First, recent research highlights potential sex‐dependent variations in bruxism prevalence, etiology, and clinical manifestations. While some studies suggest a higher prevalence of bruxism in women [133, 134, 135], others report no significant sex differences when controlling for confounding factors [136]. These discrepancies may stem from methodological variations or differences in study populations.

Hormonal influences, particularly in women, have been proposed as a contributing factor. A study [137, 138, 139] found that fluctuations in estrogen and progesterone may exacerbate sleep bruxism, potentially explaining why some studies report higher rates in females. Additionally, psychological factors such as stress and anxiety, which are often more frequently reported in women, could further contribute to this disparity [140, 141].

Conversely, studies [29, 142] observed that bruxism prevalence varies more with age than sex, suggesting that hormonal changes across the lifespan (e.g., menopause, andropause) may play a role rather than sex alone. The current literature presents mixed findings on sex‐dependent differences in bruxism. While hormonal and psychological factors may contribute to higher bruxism rates in women, the lack of consistent evidence suggests that sex alone is not a definitive predictor. Future studies should employ standardized diagnostic criteria and larger, sex‐balanced cohorts to clarify these associations.

Second, the relationship between malocclusion and bruxism remains inconclusive, as recent studies suggest only a weak or negligible association, with greater emphasis on neurophysiological and psychological factors [143, 144]. Additionally, variations in diagnostic criteria (e.g., self‐report vs. polysomnography) and heterogeneous study populations may affect the generalizability of findings. Future research should prioritize standardized methodologies and longitudinal designs to clarify these unresolved aspects.

## Author Contributions

Kyle Morris, Karima Ait‐Aissa, Amal M. Sahyoun, Qi Wang, Ammaar Abidi, Modar Kassan contributed to conception and design, drafted the manuscript, critically revised the manuscript, gave final approval, and agree to be accountable for all aspects of the work ensuring integrity and accuracy.

## Conflicts of Interest

The authors declare no conflicts of interest.

## Data Availability

Data sharing is not applicable to this article as no datasets were generated or analyzed during the current study.
